# Lipid Analysis of Fracture Hematoma With MALDI-MSI: Specific Lipids are Associated to Bone Fracture Healing Over Time

**DOI:** 10.3389/fchem.2021.780626

**Published:** 2022-03-03

**Authors:** Rald V. M. Groven, Sylvia P. Nauta, Jane Gruisen, Britt S. R. Claes, Johannes Greven, Martijn van Griensven, Martijn Poeze, Ron M. A. Heeren, Tiffany Porta Siegel, Berta Cillero-Pastor, Taco J. Blokhuis

**Affiliations:** ^1^ Division of Traumasurgery, Department of Surgery, Maastricht University Medical Center, Maastricht, Netherlands; ^2^ Department of Cell Biology-Inspired Tissue Engineering, MERLN Institute for Technology-Inspired Regenerative Medicine, Maastricht University, Maastricht, Netherlands; ^3^ Division of Imaging Mass Spectrometry, Maastricht MultiModal Molecular Imaging (M4i) Institute, Maastricht University, Maastricht, Netherlands; ^4^ Department of Orthopedic Surgery and Traumasurgery, Maastricht University Medical Center, Maastricht, Netherlands; ^5^ Department of Orthopaedics, Trauma and Reconstructive Surgery, University Hospital RWTH Aachen, Aachen, Germany; ^6^ NUTRIM, School for Nutrition and Translational Research in Metabolism, Maastricht University, Maastricht, Netherlands

**Keywords:** fracture hematoma, fracture healing, MALDI-MSI, sample preparation, lipids

## Abstract

**Background:** Fracture healing is a complex process, involving cell-cell interactions, various cytokines, and growth factors. Although fracture treatment improved over the last decades, a substantial part of all fractures shows delayed or absent healing. The fracture hematoma (fxh) is known to have a relevant role in this process, while the exact mechanisms by which it influences fracture healing are poorly understood. To improve strategies in fracture treatment, regulatory pathways in fracture healing need to be investigated. Lipids are important molecules in cellular signaling, inflammation, and metabolism, as well as key structural components of the cell. Analysis of the lipid spectrum in fxh may therefore reflect important events during the early healing phase. This study aims to develop a protocol for the determination of lipid signals over time, and the identification of lipids that contribute to these signals, with matrix-assisted laser desorption/ionization mass spectrometry imaging (MALDI-MSI) in fxh in healthy fracture healing.

**Methods:** Twelve fxh samples (6 porcine; 6 human) were surgically removed, snap frozen, sectioned, washed, and analyzed using MALDI-MSI in positive and negative ion mode at different time points after fracture (porcine: 72 h; human samples: range 1–19 days). A tissue preparation protocol for lipid analysis in fxh has been developed with both porcine and human fxh. Data were analyzed through principal component- and linear discriminant analyses.

**Results:** A protocol for the preparation of fxh sections was developed and optimized. Although hematoma is a heterogeneous tissue, the intra-variability within fxh was smaller than the inter-variability between fxh. Distinctive *m/*z values were detected that contributed to the separation of three different fxh age groups: early (1–3 days), middle (6–10 days), and late (12–19 days). Identification of the distinctive *m/*z values provided a panel of specific lipids that showed a time dependent expression within fxh.

**Conclusion:** This study shows that MALDI-MSI is a suitable analytical tool for lipid analysis in fxh and that lipid patterns within fxh are time-dependent. These lipid patterns within fxh may serve as a future diagnostic tool. These findings warrant further research into fxh analysis using MALDI-MSI and its possible clinical implications in fracture treatment.

## Introduction

Fracture healing is a complex process in which a great variety of cells, signaling molecules, and cellular signaling pathways are involved (Claes et al., 2012). Although fracture treatment has advanced greatly over the past decades, a substantial portion of fractures still suffers from impaired healing in the form of delayed healing or non-union (Claes et al., 2012; Bastian et al., 2016a). Treatment of these complications often consists of multiple surgical interventions and, even if the interventions are successful, a long trajectory of rehabilitation (Volpin and Shtarker, 2014). Therefore, insights in fracture healing processes at an early stage may help to prevent or treat these complications (Dimitriou et al., 2005; Kolar et al., 2010; Wang et al., 2017).

The fracture healing process can be divided into three main phases: inflammation, bone repair, and bone remodeling (Bastian et al., 2016a). The first phase starts immediately after the fracture occurs, with the formation of the fracture hematoma (fxh). The fxh is considered to play a pivotal role in proper fracture healing, since it initiates the inflammatory response and creates a microenvironment to which a variety of cells is attracted by means of chemotaxis. Studies have shown that the removal or debridement of the fxh as well as an overshoot in the inflammatory response can induce adverse effects on fracture healing (Park et al., 2002; Claes et al., 2012; Bastian et al., 2016a; Loi et al., 2016; Schell et al., 2017). Cytokines and growth factors within the fxh facilitate the influx of inflammatory- and mesenchymal stem cells and regulate capillary growth, which is necessary to replace extracellular matrix with granulation tissue (Kolar et al., 2010; Claes et al., 2012; Schell et al., 2017). Therefore, the molecular environment in fxh changes based on this cellular trafficking (Kolar et al., 2010; Bastian et al., 2016b; Ghiasi et al., 2017).

During the following phases of fracture healing, the fxh acts as a basis for the formation of granulation tissue, also known as callus, which will gradually ossify as time progresses to bridge the fracture gap. Being an important initiator of the fracture healing cascade, investigating the molecular environment of the fxh at different time points after trauma could provide more insight into early fracture healing processes. Mass spectrometry offers potential advantages in understanding these dynamic molecular patterns and their regulatory effects on fracture healing as well as their possible complications.

Matrix-assisted laser desorption/ionization mass spectrometry imaging (MALDI-MSI) is a suitable imaging modality that allows for the untargeted, spatially resolved detection of a variety of molecules in tissue sections. The applicability of this technique to many research fields derives from its intrinsic property to detect a broad range of molecular classes, such as lipids or proteins, in a single experiment, without prior knowledge or hypothesis on the composition of a sample (Chughtai and Heeren, 2010).

**FIGURE 1:**
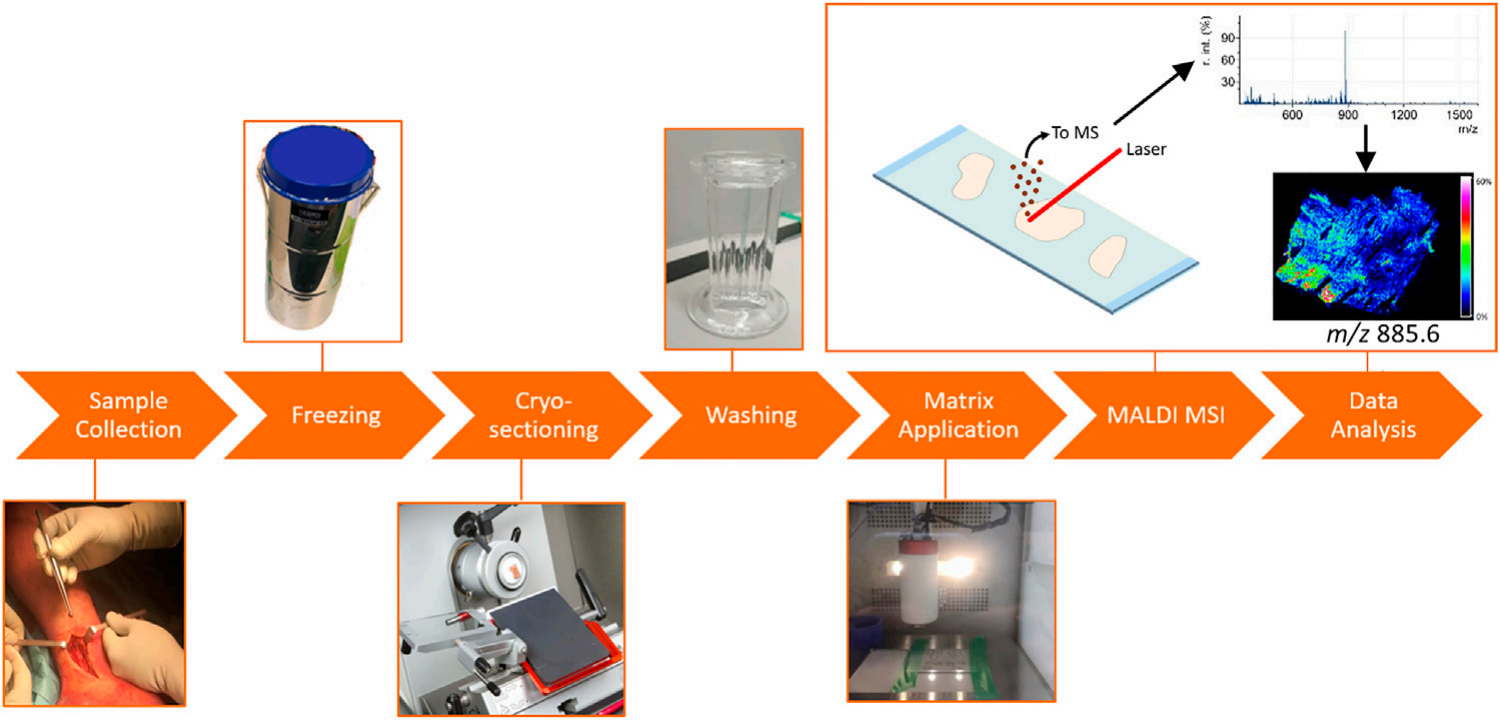
General MALDI-MSI workflow, applied to fracture hematoma analysis. The fxh was surgically removed and snap frozen in liquid nitrogen. The fxh was cryo-sectioned and thaw mounted on ITO slides. The slides were washed by submerging them in either ammonium formate or acetone. Afterwards, norharmane was homogenously sprayed onto the slides. The coated slides were analyzed by MALDI-MSI after which the data were processed and visualized.

A schematic overview of the MALDI-MSI workflow is depicted in Figure 1. A tissue section is covered with a matrix, which co-crystallizes with the analyte molecules. The matrix-analyte mixture absorbs the energy of the focused laser beam hitting the sample surface. This results in the local desorption and ionization of the analytes into the gas phase. The analysis of these ions with a mass spectrometer provides a mass spectrum, which depicts their mass-to-charge ratio (*m/z*). The analysis of mass spectra of multiple laser spots (or pixels) allows for the determination of the spatial distribution of certain molecules in the sample (Glish and Vachet, 2003; Murphy et al., 2009; Chughtai and Heeren, 2010; Watrous et al., 2011; Norris and Caprioli, 2013; Aichler and Walch, 2015). Histological staining of the same section after MSI enables the correlation of the detected molecules to a certain anatomical region within the sample.

To our knowledge, MALDI-MSI has not yet been reported for the analysis of fracture healing processes. Using MALDI-MSI to analyze fxh at different time points during the fracture healing process could help to understand changes in molecular patterns throughout different phases of fracture healing. Lipids are suitable target molecules for the examination of potential use and application of MALDI-MSI for fxh analysis since they are key molecules in cellular signaling, inflammation, and metabolism, as well as important structural components of the cell (Schwamborn and Caprioli, 2010; Li et al., 2014; Øyen et al., 2017; Chiurchiù et al., 2018; Matsumoto et al., 2019; Alekos et al., 2020; Kang et al., 2020).

In this study, we used MALDI-MSI to investigate the lipid signature and its spatial distribution in fxh samples taken at different time points between trauma and surgery. A sample preparation procedure was developed to improve lipid signal intensities. One of the key challenges in this sample preparation was the removal of blood from the fxh with a washing step, since it contains large quantities of heme. Heme is easily ionized and therefore negatively affects the ionization of lipids and other molecules, a process called ion suppression. The washing step should improve lipid signal intensities without delocalization. Furthermore, the intra-variability of the detected molecules within the fxh was evaluated to establish if differences in molecular patterns occur based on their specific location within the fxh. Thus, the aims of this study are to: 1) Develop a methodology for analysis of fxh with MALDI-MSI, 2) Investigate the intra-variability of the molecular profile within fxh, and 3) Identify fxh time-dependent molecular patterns between the fxh.

## Materials and Methods

### Materials

Ammonium formate, norharmane, Mayer’s hematoxylin, and eosin were purchased from Sigma Aldrich (St. Louis, MO, USA). Acetone (HPLC grade), chloroform (HPLC grade), ethanol (HPLC grade), n-hexane (HPLC grade), methanol (ULC/MS-CC/SFC grade), and deionized water (ULC/MS-CC/SFC grade) were purchased from Biosolve B.V. (Valkenswaard, Netherlands).

### Samples—Fracture Hematoma

Fxh were obtained from pigs for method development and investigation of the intra-variability. Fxh were obtained from humans for the method validation and the investigation of time-dependent molecular patterns. An overview of the fxh tissue and age used for the different objectives can be found in Table 1. The fxh age is defined as the number of days between the bone fracture and surgical intervention.

**TABLE 1:** Overview of fxh tissues and ages used for the different objectives.

Objective	fxh tissue	fxh age
Tissue washing comparison	1 Human	3 days
	1 Porcine	3 days
Intra-hematoma variability	4 Porcine	3 days
Detection of molecular patterns	2 Human	2 days
	2 Human	9 days
	2 Human	19 days

#### Porcine fxh

Porcine fxh were collected from closed tibia shaft fractures that were created during animal experiments, as previously published by Guo et al. (2020) In short, a multi-trauma model was used to study hematological and chemical profiles in a porcine model of severe multi-trauma over a time period of 72 h after trauma. During the experiments, all animals were kept sedated and fxh were harvested 72 h after fracture induction. All procedures were approved by the animal care and use office of the state of Nordrhein-Westfalen (approval number: LANUV AZ 81–02.04.2017.A412).

#### Human fxh

The human fxh were collected during open surgical reduction and internal fixation of metaphyseal long bone fractures. For the detection of molecular patterns, samples were gathered from male patients with metaphyseal bone fractures, without further known comorbidities, that showed uneventful fracture healing, with a mean age of 51 ± 20 years. Due to patient-specific reasons, fracture surgery was performed at different time points after trauma which enables harvesting of a range of fxh of different ages (2–19 days). The Medical Ethical Committee of the MUMC+ approved this study (approval number: MEC 16-4-251).

### General Sample Preparation and MALDI-MSI Protocol

The workflow for the sample preparation and MALDI-MSI analysis of fxh can be described in six steps (Figure 1). H&E staining was performed after MALDI-MSI analysis. The general protocol was used for the different experiments with minor adaptions when specified.

Sample collection and freezing: The fxh were harvested and snap-frozen in aluminum containers dipped in liquid nitrogen to stop cellular biological processes (Goodwin, 2012). The fxh were stored at −80°C until further use. The containers were put on ice when transferring for a short period of time.

Cryo-sectioning: Indium tin oxide (ITO) slides were cleansed for 10 min each by sonicating them in n-hexane and ethanol consecutively. The fxh were cryo-sectioned at 12 µm using a Leica CM1860 UV cryostat (Leica microsystems, B.V., Amsterdam, the Netherlands) between −22 and −24°C. The sectioned fxh were thaw mounted on ITO slides (Delta Technologies, Colorado, USA). Afterwards, the slides with fxh sections were dried with nitrogenous gas. The slides were stored at −80°C, until further use.

Washing: The ITO slides with tissues sections were submerged for a defined amount of time (15–120 s) in either ammonium formate or acetone. After submersion, the slides were dried by nitrogen gas. More details about this step can be found in the paragraph “Comparison of different tissue washing methods”.

Matrix application: In this study, 7 mg/ml norharmane in 2:1 chloroform and methanol (v/v) was applied for the extraction of lipids. This matrix solution was sonicated for 15 minutes and applied to the slides using an HTX TM sprayer (HTX technologies, LC, North Carolina, USA). Ten layers of matrix were applied with a drying time of 30 s between each layer using a nozzle temperature of 30°C and a flow rate of 0.12 μL/min. The velocity was set at 1,200 mm/min with a track spacing of 3 mm.

MALDI-MSI: Experiments were performed with a RapifleX MALDI Tissuetyper mass analyzer system (Bruker, Bremen, Germany) in reflector mode. The data were acquired at a 50 µm by 50 µm raster size with 200 laser shots/pixel at a laser frequency of 10 kHz. Positive and negative ion mode spectra were acquired to analyze lipids over a mass range of *m/z* 340–1,200 and 340–1,600, respectively. The system was calibrated with red phosphorus before acquisition.

Data analysis: FlexImaging v4.1 (Bruker Daltonik GmbH, Bremen, Germany) was used to visualize the molecular distributions. SCiLS lab 2016b (SCiLS GmbH, Bremen, Germany) was used for data analysis and data conversion. Principal component analysis—linear discriminant analysis (PCA-LDA) was performed and visualized with an in-house-built ChemomeTricks toolbox for MATLAB (version 2014a, The MathWorks, Natick, USA) to compare different groups.

H&E staining: After the MSI analysis, the matrix was removed from the slides with 70% ethanol. The slides were hematoxylin-eosin (H&E) stained. The stained slides were scanned with a M8 Microscope and Scanner (PreciPoint, Freising, Germany) at ×20 magnification and co-registered with the MALDI-MSI distribution images.

### Comparison of Different Tissue Washing Methods

Different tissue washing methods were compared to increase lipid signal intensities after washing of heme. In addition, the possible delocalization of molecules caused by the washing of the lipids was assessed. Slides with porcine or human fxh were submerged in ammonium formate (50 mM in water, pH 7) or acetone. In-between washing steps and at the end, slides were dried with a gentle flow of nitrogen gas. Unwashed samples served as a control. An overview of the different washing methods compared for negative and positive ion modes can be found in Table 2. The number of washing methods tested for positive ion mode was reduced based on the results in negative ion mode, as the longest washing methods resulted in reduced lipid intensity and increased lipid delocalization. Three technical replicates were acquired per washing method. The remaining tissue preparation protocol and analyses were as described above in the general methods.

**TABLE 2:** Overview of the different washing methods that were applied to the fxh, including the polarity.

Washing solvent	Washing time	Negative ion mode	Positive ion mode
Ammonium formate	30 s	X	X
	15 s twice	X	X
	30 s twice	X	-
	15 s four times	X	-
Acetone	30 s twice	X	X
	30 s four times	X	-
No-wash (control)	-	X	X

Comparison of the washing methods was done based on the mean signal-to-noise (S/N) values of ten selected ion peaks and the heme peak (*m/z* value based on literature). In addition, the ratios of the intensity of selected *m/z* values over the heme ion intensity (M/H) were compared. The *m/z* values were selected based on the highest intensities (excluding isotopes) and to cover the mass range of *m/z* 650–950. The optimal washing method was selected based on the combination of high ratios, adequate blood removal, and minimal lipid delocalization.

### Intra-variability of fxh

A comparison of the molecular profiles of the outside and center of the fxh was performed to study the heterogeneity of the samples. For this purpose, multiple slides were created with three consecutive sections of the outside and the center for four porcine fxh (see Supplementary Figure S1). The sections for the outside were sectioned as close to the border of the sample as possible after minimal trimming. The center of the fxh was determined based on visual observation of size and shape, as the center differs distinctively for each fracture hematoma due to their differences in size and shape. The remaining tissue preparation protocol and analyses were as described above in the general method.

### Identify fxh Age-Dependent Molecular Patterns

One of the objectives was to compare the molecular profiles of human fxh of different fxh ages. The selected fxh ages were two, nine, and 19 days after bone fracture to represent different time points in the early stages of the fracture healing cascade. Per sample at least five technical replicates were acquired. The slides were used for acquisition in negative and positive ion mode. The remaining tissue preparation protocol and analyses were as described above in the general method.

The different fxh ages were compared in both negative and positive ion mode using PCA-LDA. This analysis defines discriminant functions (DFs) that maximize the variance between classes while minimizing the variance within classes. The DF-score plots show which groups can be separated using that DF while the scaled loadings plot provides the corresponding *m/z* values. The scaled loadings are a combination of the intensity of the *m/z* value and the extent to which the molecule contributes to the separation of classes across the specified discriminant function. The scaled loadings on the positive side contribute more to the class at the positive side of the DF score and vice versa.

### Lipid Identification

Molecules of interest were determined based on the PCA-LDA analysis of human fxh of different fxh ages. These molecules of interest are identified based on the highest scaled loadings for the different classes. Background peaks, matrix clusters, and isotopes were removed from the lists of *m/z* values with high scaled loadings. The molecules were identified by MS/MS analysis using collision-induced dissociation (CID) to fragment molecules of interest. Data for lipid identification were acquired using a data-dependent acquisition (DDA)-imaging method, as described previously (Ellis et al., 2018). Shortly, MSI and MS/MS data were acquired on an Orbitrap Elite hybrid ion trap mass spectrometer (Thermo Fisher Scientific GmbH, Bremen, Germany) using a stage step size of 25 × 50 μm^2^. MSI (MS^1^) data of *m/z* 300–2000 were acquired at a nominal mass resolution of 240,000 (at *m/z* 400) using an injection time of 250 ms for both positive and negative ion mode. In parallel, the MS/MS data were acquired using the ion trap with an isolation window of 1 Da; a normalized collision energy of 30.0 (manufacturer units) with an activation q value of 0.17 in positive ion mode, and 38.0 and 0.25 in negative ion mode, respectively. Data analysis and lipid assignments were performed using Thermo Xcalibur (version 4.2, Thermo Fisher Scientific) and LipostarMSI (version 1.1.0b26, Molecular Horizon, Bettona, Italy) (Tortorella et al., 2020).

## Results

### Comparing Different Tissue Washing Methods

#### Porcine fxh

**FIGURE 2:**
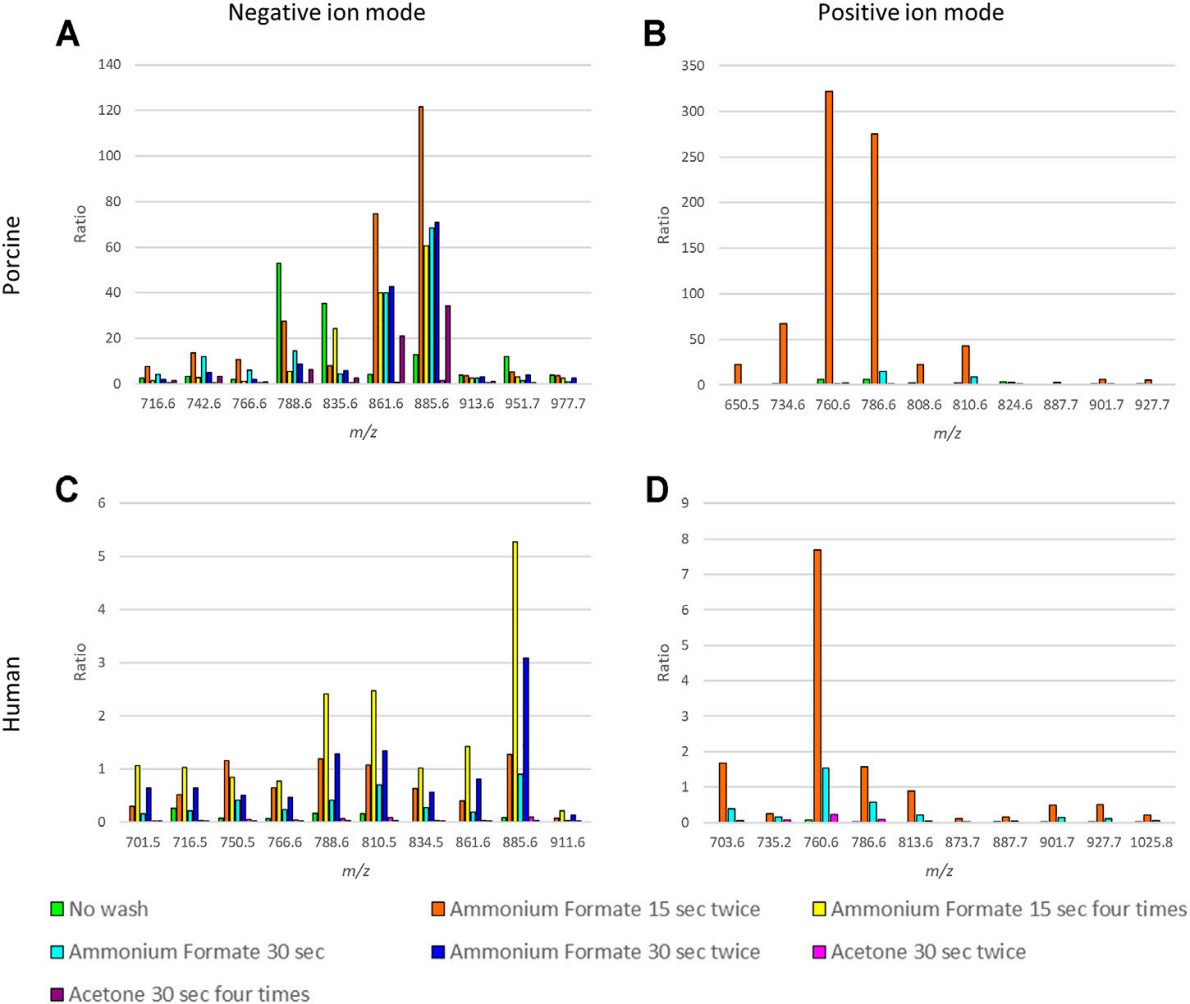
Comparison of the ratios between heme and selected *m/*z values for different washing methods for negative and positive ion mode for porcine and human fracture hematoma. The ratios represent the ion intensities of selected *m/z* values over the heme ion intensity. The *m/z* values were selected based on the highest intensities (excluding isotopes) and to cover the mass range of lipids (*m/z* 650–950). **(A)** Ratios for 10 *m/z* values for the six washing methods and the control (no washing) for porcine fxh in negative ion mode. **(B)** Ratios for 10 *m/z* values for the three washing methods and the control (no washing) for porcine fxh in positive ion mode. **(C)** Ratios for 10 *m/z* values for the six washing methods and the control (no washing) for human fxh in negative ion mode. **(D)** Ratios for 10 *m/z* values for the three washing methods and the control (no washing) for human fxh in positive ion mode.

The ratios between the mean intensities of selected *m/z* values and heme, as well as the S/N values of the selected *m/z* values of porcine fxh in both ion modes, were increased after washing the sections in either ammonium formate or acetone (Figures 2A,B, Supplementary Table S1A,B).

In negative ion mode, submerging the sections in ammonium formate for 15 seconds twice resulted in the highest ratios for most *m/z* values, as seen in Figure 2A. Washing the sections in acetone for 30 seconds twice enhanced the S/N value for heme compared to no wash, while for the other washing methods the heme S/N value decreased (Supplementary Table S1A). The highest S/N values for most of the selected *m/z* values were obtained with washing in ammonium formate for 30 seconds once or 15 seconds twice.

In positive ion mode, submerging the sections in ammonium formate for 15 seconds twice resulted in the highest ratios for most *m/z* values (Figure 2B). Only washing twice with ammonium formate for 15 seconds decreased the S/N value for heme compared to no wash (Supplementary Table S1B). For the other methods, the S/N value of heme increased. The highest S/N values for most of the selected *m/z* values were obtained with washing in ammonium formate for 30 seconds once or 15 seconds twice.

#### Human fxh

MSI results of human fxh also improved in both ion modes. The ratios between the mean intensities of selected *m/z* values and heme as well as the S/N values of the selected *m/z* values were increased after washing the sections in either ammonium formate or acetone, as for the porcine samples (Figures 2C,D, Supplementary Table S1C,D).

In negative ion mode, submerging the sections in ammonium formate for 15 seconds four times resulted in the highest ratios for most *m/z* values (Figure 2C). Washing the sections in ammonium formate for 30 seconds twice or 15 seconds four times resulted in a decreased S/N value of heme compared to unwashed controls, while the other washing methods resulted in an increased heme S/N value (Supplementary Table S1C). The highest S/N values for all the selected *m/z* values were obtained with washing in ammonium formate for 30 seconds once or 15 seconds twice, despite the S/N ratios being the highest for ammonium formate for 15 seconds four times.

In positive ion mode, submerging the sections in ammonium formate for 15 seconds twice resulted in the highest ratios for all of the *m/z* values (Figure 2D). All the washing methods resulted in an increased heme S/N value compared to no wash, but this increase was the smallest for an ammonium formate wash of 15 seconds twice (Supplementary Table S1D). The highest S/N values for all the selected *m/z* values were obtained with washing in ammonium formate for 30 seconds once or 15 seconds twice.

#### Delocalization

**FIGURE 3:**
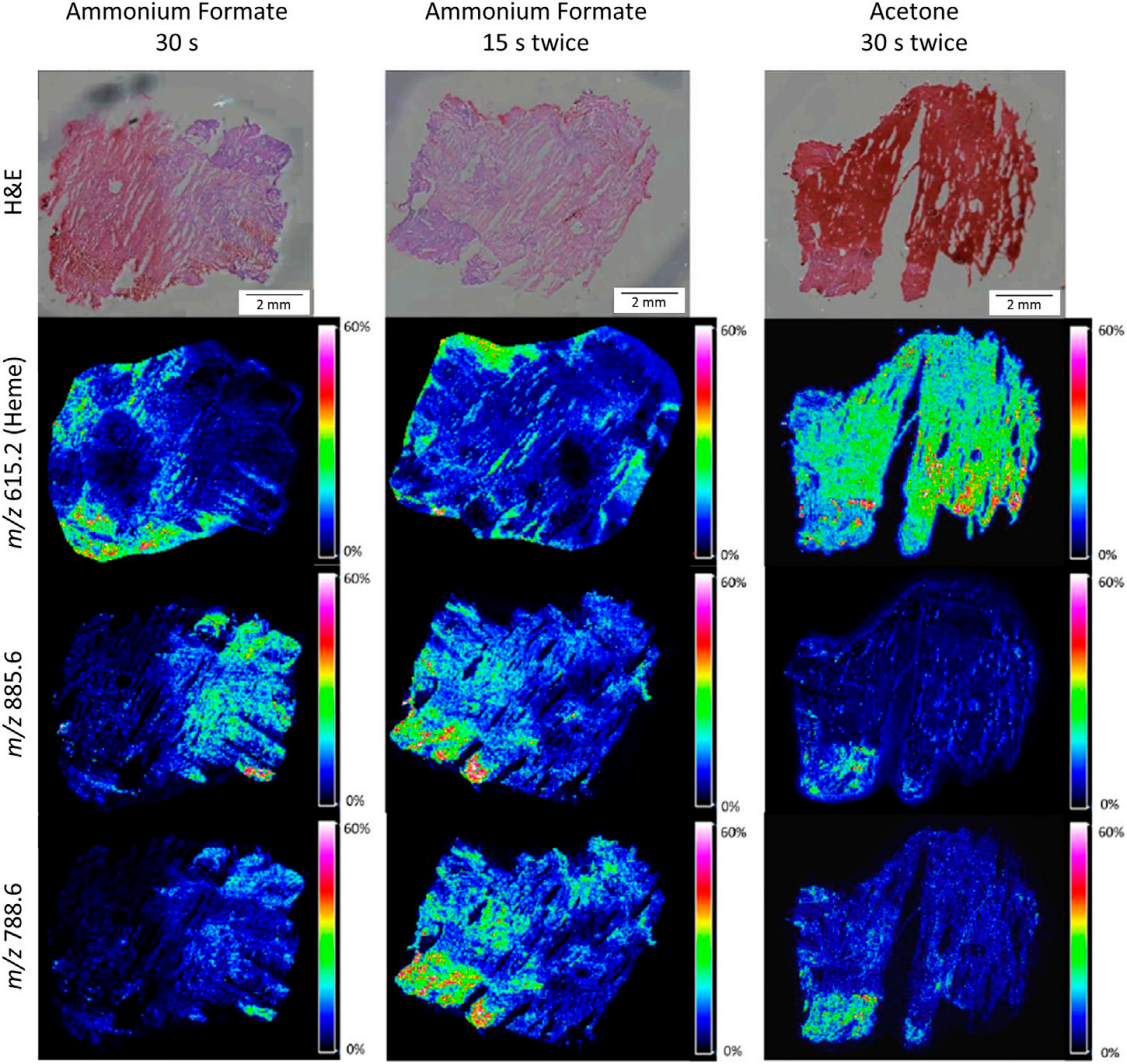
H&E stained and MALDI-MSI distribution images for three different washing methods for human fxh. MALDI-MSI images are shown for the distribution of heme (*m/z* 615.2) and lipids with *m/z* values 885.6 and 788.6 in negative ion mode for the washing methods: ammonium formate for 30 s (left column), ammonium formate for 15 s twice (middle column), and acetone for 30 s twice (right column). All shown intensities are total ion current (TIC) normalized.

Figure 3 and Supplementary Figure S2 show example images of MALDI-MSI distribution images for different washing methods for selected washing methods. The ammonium formate wash shows less delocalization than the acetone wash (Figure 3 and Supplementary Figure S2). Almost no heme was present within the fxh tissue for the tissue washing method with ammonium formate, in contrast to washing with acetone (Figure 3 and Supplementary Figure S2). Nevertheless, the intensity of heme is relatively high outside the tissue for the ammonium formate washing methods, which is attributed to the removal of the heme from the tissue with these methods. Increased delocalization was observed with increasing washing time, for example, the delocalization was larger for ammonium formate wash 15 s four times than for 15 s twice. In addition, it was observed that the delocalization for the selected *m/z* values was minimal and the highest intensities were present within the tissue for the ammonium formate wash.

Taking the above-mentioned results into account, the optimal fxh washing method is the ammonium formate wash for 15 s twice based on the increased intensity of the selected *m/z* values, the decreased heme intensity, and minimum delocalization of the molecules of interest. This washing method was further applied throughout this study.

### Intra-Variability of fxh

Fxh is a very heterogeneous tissue due to the presence of different cell types, which affects the molecular profile as well. The molecular profiles of the outside and center of fxh are compared to examine the intra-variability of this profile throughout the fxh. This is important to prevent bias based on sampling location within a fxh.

**FIGURE 4:**
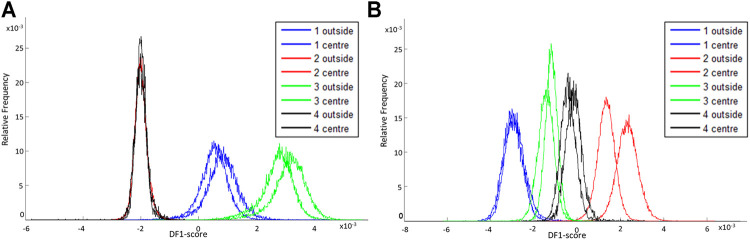
DF-1 scores for comparison of the intra-variability of fracture hematoma for negative and positive ion mode. The DF-1 score explains the biggest variance in the data set as determined by a PCA-LDA of the mass spectra of the outside and center sections of different porcine fxh. **(A)** DF-1 score for the outside and center sections of four porcine fxh in negative ion mode. **(B)** DF-1 score for the outside and center sections of four porcine fxh in positive ion mode.

Four porcine fxh were used to determine if the molecular profiles differed based on the sampling location within the fxh, which reflects the intra-variability of the fxh. Figure 4A shows that molecular profiles of the outside and center of fxh cannot be distinguished in negative ion mode, as the graphs for the outside and center section per sample mostly overlap. For positive ion mode, the same holds, although the intra-variability for sample 2 is bigger than for the other samples, as those graphs overlap less (see Figure 4B). The DF-2 scores (see Supplementary Figure S3) show the same trend for both negative and positive ion mode. In general, the results from the PCA-LDA analysis show that the inter-variability between samples is higher than the intra-variability within a sample.

### Identify fxh Age-Dependent Molecular Patterns

Human fxh of different fxh ages (2, 9, and 19 days) were analyzed to compare the differences in molecular profiles at an early, middle, and late stage during fracture healing. Changes in the molecular profiles can help to improve the understanding of molecular changes during bone fracture healing.

#### Negative Ion Mode

**FIGURE 5:**
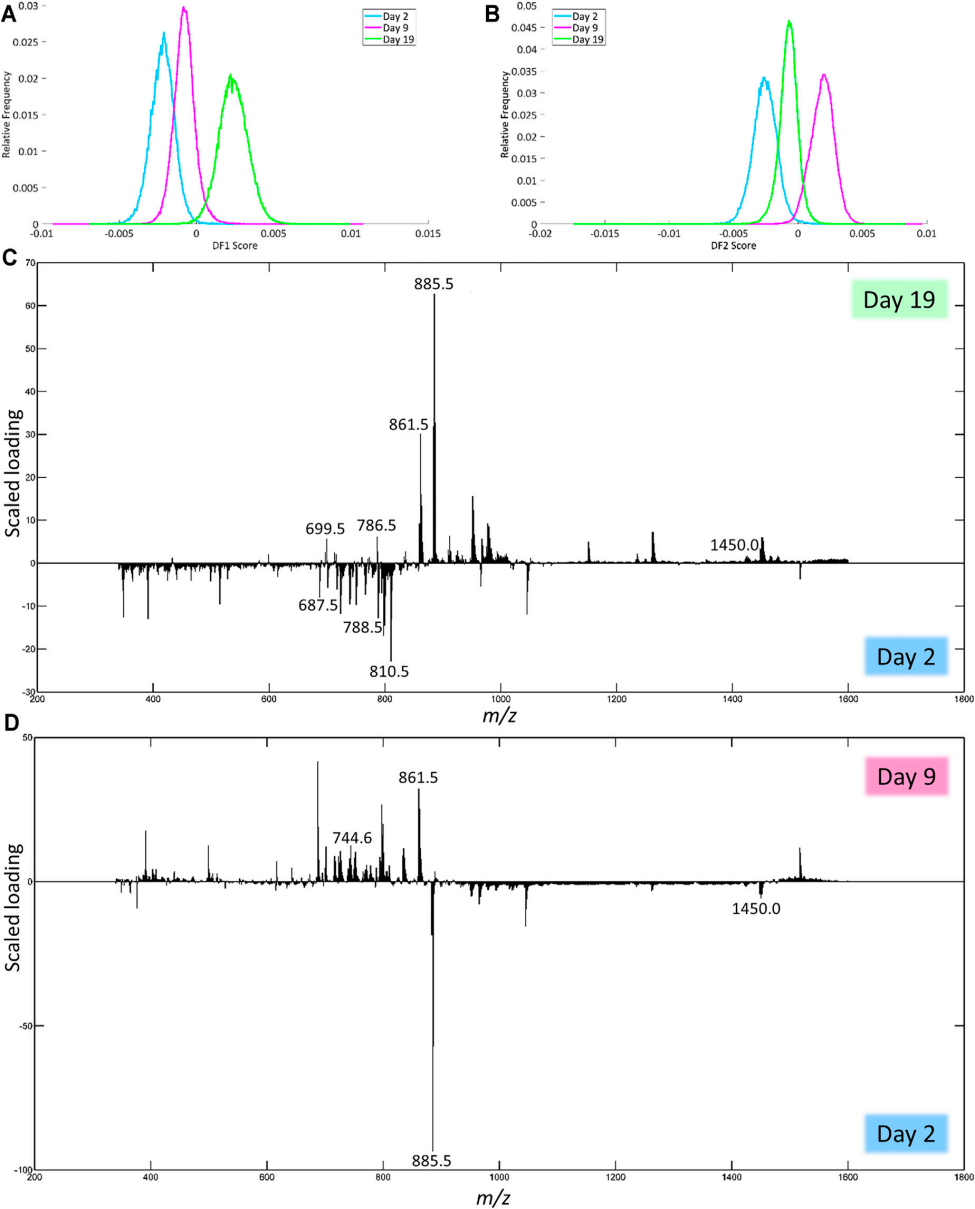
Results of the PCA-LDA of the human fxh of different fxh age (2, 9, and 19 days) for negative ion mode. DF-scores and their corresponding scaled loading plots are shown. The scaled loading is a combination of the intensity of the *m/z* value with how much the molecule contributes to the separation of classes across the specified discriminant function. The positive side for the DF-score plot is related to the positive side of the scaled loading plots. This indicates that the *m/z* values at a certain side have a higher contribution in the class at that side. **(A)** DF-1 score representing the first discriminant function, which corresponds to the highest variance in the dataset. **(B)** The DF-2 score representing the second discriminant function. **(C)** Scaled loading plot of the full mass range for DF-1. **(D**) Scaled loading plot of the full mass range for DF-2.

In negative ion mode, DF-1 explains 1.47% of the total variance of the dataset and can be used to separate day 2 from day 19 (Figure 5A). Figure 5C shows the corresponding scaled loading plot. In the range from *m/z* 600 to 830, the peaks are more distinctive of day 2, while the peaks in the range *m/z* 830 to 1,000 and some peaks in the mass range above that more distinctive of day 19. The results of the identification of the peaks with the highest scaled loadings can be found in Table 3 (labeled with DF-1). For the separation between day 2 and day 19, certain phosphatidylinositols (PIs) and cardiolipins (CLs) are more characteristic of day 19, while a phosphatidylethanolamine (PE) is more characteristic of day 2. Phosphatidic acids (PAs) and phosphatidylserines (PSs) are present on both day 2 and 19, but the specific fatty acid chain composition and degree of saturation differ between both classes.

**TABLE 3:** Lipid assignments based on MS/MS data and high mass resolution experiments in negative ion mode. For each assignment, the *m/z* value obtained with the high mass resolution mass spectrometer (Orbitrap Elite), the lipid assignment, the detected ion, the ppm error, the condition to which the *m/z* value contributes, the DF, and the corresponding scaled loading are provided.

*m/z* value	Assignment	Ion	Δ ppm error	Condition	DF
687.54	SM 16:0_18:1; O2	[M-CH3]^-^	0.5	Day 2	DF-1
699.50	PA 18:0_18:2 and PA 18:1_18:1	[M-H]^-^	0.5	Day 19	DF-1
701.51	PA 18:0_18:1	[M-H]^-^	0.5	Day 2	DF-1
				Day 9	DF-2
716.52	PE 16:0_18:1	[M-H]^-^	0.7	Day 9	DF-2
723.50	PA 18:0_20:4	[M-H]^-^	0.4	Day 2	DF-1
				Day 9	DF-2
742.54	PE 18:0_18:2 and PE 18:1_18:1	[M-H]^-^	0.5	Day 9	DF-2
744.60	PE 18:0_18:1	[M-H]^-^	0.6	Day 9	DF-2
766.54	PE 18:0_20:4	[M-H]^-^	0.9	Day 2	DF-1
770.57	PE 18:0_20:2 and PE 18:1_20:1 and PE 18:2_20:0	[M-H]^-^	1.8	Day 9	DF-2
786.53	PS 18:0_18:2 and PS 18:1_18:1	[M-H]^-^	0.5	Day 19	DF-1
788.54	PS 18:0_18:1	[M-H]^-^	0.6	Day 2	DF-1
				Day 9	DF-2
810.53	PS 18:0_20:4	[M-H]^-^	0.5	Day 2	DF-1
				Day 9	DF-2
833.52	PI 16:0_18:2 and PI 16:1_18:1 and PI 16:2_18:0	[M-H]^-^	0.6	Day 9	DF-2
835.53	PI 16:1_18:0 and PI 16:0_18:1	[M-H]^-^	1.1	Day 19	DF-1
				Day 9	DF-2
859.53	PI 18:1_18:2	[M-H]^-^	0.1	Day 19	DF-1
861.54	PI 18:0_18:2 and PI 18:1_18:1	[M-H]^-^	0.6	Day 19	DF-1
				Day 9	DF-2
863.56	PI 18:0_18:1	[M-H]^-^	0.8	Day 19	DF-1
				Day 9	DF-2
883.53	PI 18:1_20:4	[M-H]^-^	0.8	Day 19	DF-1
				Day 2	DF-2
885.55	PI 18:0_20:4	[M-H]^-^	0.8	Day 19	DF-1
				Day 2	DF-2
909.55	PI 18:0_22:6	[M-H]^-^	0.9	Day 19	DF-1
911.56	PI 18:0_22:5 and PI 18:1_22:4	[M-H]^-^	1.0	Day 19	DF-1
1447.96	CL 18:2_18:2_18:2_18:2	[M-H]^-^	0.4	Day 19	DF-1
				Day 2	DF-2
1449.98	CL 18:1_18:2_18:2_18:2	[M-H]^-^	1.0	Day 19	DF-1

DF-2 explains 2.39% of the total variance and can be used to separate day 2 from day 9 (Figure 5B). The corresponding scaled loading plot (Figure 5D) shows that most of the peaks in the mass range up to *m/z* 880 are more distinctive of day 9, while most peaks above this value are more distinctive of day 2 except for the peaks just above *m/z* 1,515, which correspond to day 9. The results of the identification of the peaks with the highest scaled loadings can be found in Table 3 (labeled with DF-2). For the separation between days 2 and 9, certain PAs, PEs, and PSs are more characteristic of day 9, while certain CLs are more characteristic of day 2. PIs are present on both day 2 and 9, but the specific fatty acid chain composition and degree of saturation differ between both classes.

#### Positive Ion Mode

**FIGURE 6:**
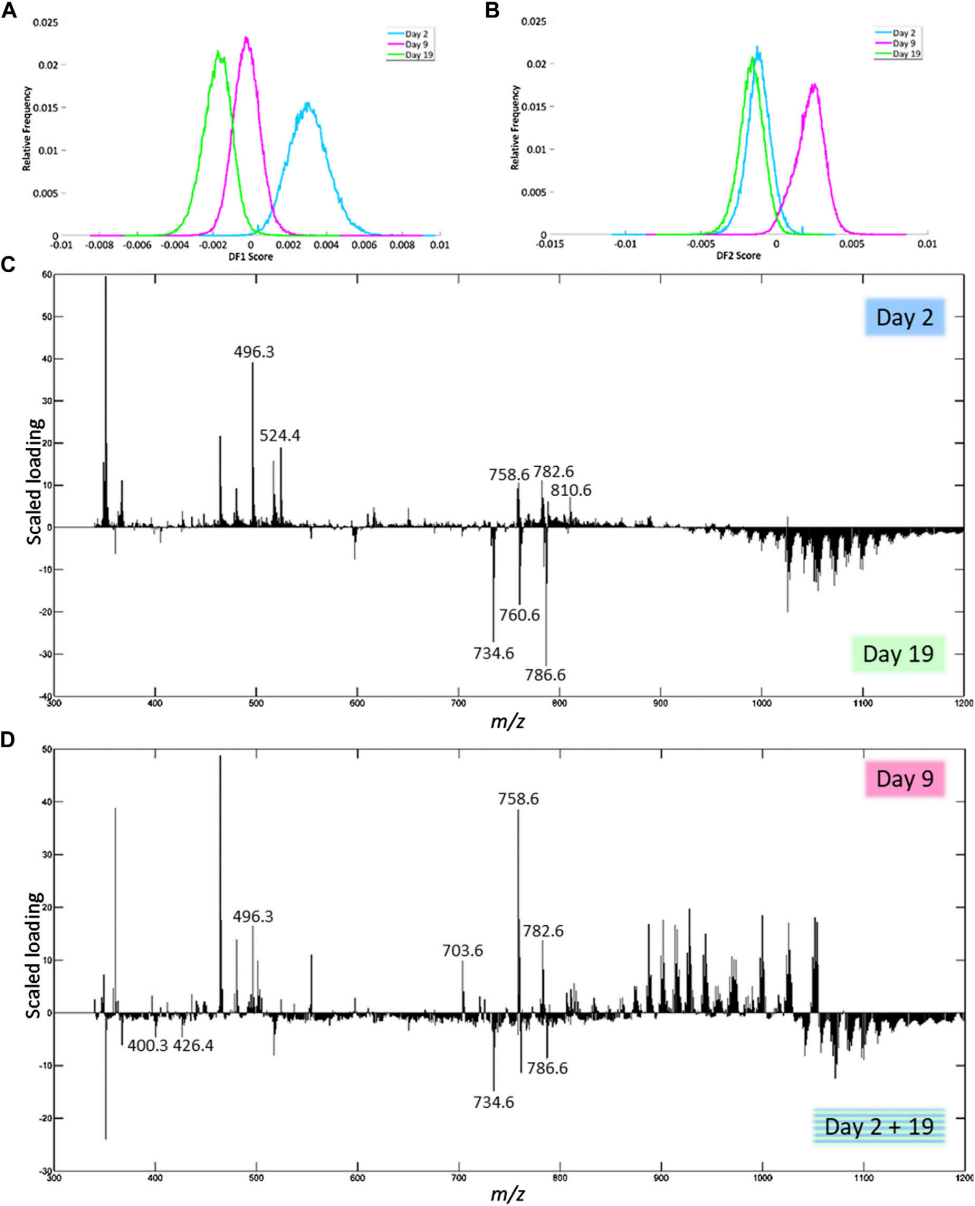
Results of the PCA-LDA of the human fxh of different fxh age (2, 9, and 19 days) for positive ion mode. DF-scores and their corresponding scaled loading plots are shown. The scaled loading is a combination of the intensity of the *m/z* value with how much the molecule contributes to the separation of classes across the specified discriminant function. The positive side for the DF-score plot is related to the positive side of the scaled loading plots. This indicates that the *m/z* values at a certain side have a higher contribution in the class at that side. **(A)** DF-1 score representing the first discriminant function, which corresponds to the highest variance in the dataset. **(B)** The DF-2 score representing the second discriminant function. **(C)** Scaled loading plot of the full mass range for DF-1. **(D)** Scaled loading plot of the full mass range for DF-2.

In positive ion mode, DF-1 explains 2.23% of the total variance of the dataset and can be used to separate day 2 from day 19 after trauma (Figure 6A). Based on the scaled loadings (Figure 6C), most of the peaks are more distinctive of day 2 up to *m/z* 925, except for a few peaks, most notably 3 clusters of peaks in the range of *m/z* 700 to 800. Most of the peaks above *m/z* 925 are more distinctive of day 19. The results of the identification of the peaks with the highest scaled loadings can be found in Table 4 (labeled with DF-1). For the separation between day 2 and day 19, it seems that certain lysophosphatidylcholines (LPCs) have a higher presence on day 2. The main class of identified lipids is phosphatidylcholines (PCs), PCs are present in both the day 2 and day 19. However, the specific fatty acid chain composition and degree of saturation differ between day 2 and day 19.

**TABLE 4:** Lipid assignments based on MS/MS and high mass resolution experiments in positive ion mode. For each assignment, the *m/z* value obtained with the high mass resolution mass spectrometer (Orbitrap Elite), the lipid assignment, the detected ion, the ppm error, the condition to which the *m/z* value contributes, the DF, and the corresponding scaled loading are provided.

*m/z* value	Assignment	Ion	Δ ppm error	Condition	DF
400.34	CAR 16:0	[M + H]^+^	1.0	Day 2 + 19	DF-2
426.36	CAR 18:1	[M + H]^+^	0.4	Day 2 + 19	DF-2
496.34	LPC 16:0	[M + H]^+^	0.7	Day 2	DF-1
				Day 9	DF-2
524.37	LPC 18:0	[M + H]^+^	1.5	Day 2	DF-1
703.57	SM 34:1; O2	[M + H]^+^	0.4	Day 9	DF-2
734.57	PC 16:0_16:0	[M + H]^+^	0.4	Day 19	DF-1
				Day 2 + 19	DF-2
758.57	PC 16:0_18:2	[M + H]^+^	0.5	Day 2	DF-1
				Day 9	DF-2
760.58	PC 16:0_18:1	[M + H]^+^	0.7	Day 19	DF-1
				Day 9	DF-2
768.59	PC O-36:4	[M + H]^+^	0.7	Day 2	DF-1
782.57	PC 16:0_20:4	[M + H]^+^	0.6	Day 2	DF-1
				Day 9	DF-2
784.58	PC 16:0_20:3 and PC 18:1_18:2	[M + H]^+^	0.7	Day 19	DF-1
786.60	PC 18:0_18:2 and PC 18:1_18:1	[M + H]^+^	0.7	Day 19	DF-1
				Day 2 + 19	DF-2
788.62	PC 18:0_18:1	[M + H]^+^	1.4	Day 2	DF-1
810.60	PC 18:0_20:4	[M + H]^+^	0.7	Day 2	DF-1

DF-2 explains 2.79% of the total variance and can be used to separate day 9 from day 2 + 19 (Figure 6B). The corresponding scaled loading plot (Figure 6D) shows that clusters of peaks are more distinctive of day 9 or day 2 + 19. For the mass range up to *m/z* 850 and from *m/*z 1,020, most peaks are related to day 2 + 19, while for the mass range *m/z* 850 to 1,020 most peaks are related to day 9. The results of the identification of the peaks with the highest scaled loadings can be found in Table 4 (labeled with DF-2). For the separation between day 9 and day 2 + 19, it seems that certain acylcarnitines (CARs) are more present at day 2 + 19, while an LPC and sphingomyelin (SM) are more present at day 9. Just as for DF-1, the main class of identified lipids is PCs, which are present on both day 9 and day 2 + 19. Again, the specific fatty acid chain composition and degree of saturation differ between day 9 and day 2 + 19.

## Discussion

In this study, the use of MALDI-MSI as a tool to examine the molecular events in fxh was explored. A methodology was developed for the analysis of fxh with MALDI-MSI with a focus on defining the optimal washing method. The intra-variability of the molecular profile in fxh was shown to be smaller than the inter-variability using this method. Lastly, fxh age-dependent lipid patterns within the fxh were identified. These age-dependent lipid patterns might be applied in predicting fracture healing outcome in the future.

### Tissue Preparation

A two-step washing protocol with ammonium formate followed by an intermediary drying step is most effective in reducing the heme signal, minimizing lipid delocalization, and enhancing lipid signal intensities. Our results are in line with the study by Angel et al. (2012), which shows that longer washing times go hand in hand with increased lipid delocalization (Angel et al., 2012).

In general, most molecular intensities were enhanced after tissue washing with either ammonium formate or acetone, compared to no tissue washing at all. This result is in line with the study by Seeley et al. (2008), which also showed enhanced heme signal intensities after tissue washing with acetone as compared to not washing tissue sections (Seeley et al., 2008). In this study, acetone proved to be less effective, as acetone tissue washing resulted in a higher heme signal as compared to ammonium formate tissue washing or no washing. Additionally, more residual heme was located within the tissue after acetone wash in comparison to the ammonium formate wash.

### Fracture Hematoma Intra-Variability

For the sample location of the fxh, it was shown that the intra-variability within a fxh was smaller than the inter-variability between fxh based on DF-1. No marked regions within the fxh could be identified that exhibit a specific function based on their spatial distribution. This is in line with expectations since the fxh is a very dynamic and diverse environment in which, depending on the phase of the fracture healing process, a great variety of cells is present (Kolar et al., 2010; Claes et al., 2012).

### Comparison of Human fxh of Different fxh Age

Using MALDI-MSI, distinct lipid patterns were identified in fxh samples that were indicative of fxh age. Five lipid classes in negative ion mode and four lipid classes in positive ion mode showed to contribute most to differentiating the three age-based fxh groups. Herein, we focus on the lipid classes with the highest differentiating contribution.

#### Lipid Patterns in Negative Ion Mode

In negative ion mode, days 2 and 19 could predominantly be separated by the relatively higher presence of PIs and CLs on day 19 as compared to a higher presence of PEs on day 2 (Table 3).

PIs serve as precursors of signaling molecules within both the Inositol-3-phosphate/diaglycerol (IP3/DAG), as well as the phosphatidylinositol-3-Kinase/protein kinase B (PI3K/AKT) signaling pathway and assist in the effective regulation of angiogenesis (Davies et al., 2019). Regarding bone formation, another important aspect is the recruitment and release of calcium through the binding of IP3 to the endoplasmatic reticulum. Calcium release results in a positive feedback loop on the production of DAG, which is counteracted by diaglycerol kinase due to the conversion to PA, a key molecule in the mammalian target of rapamycin (mTOR) signaling pathway, as described below (Antal and Newton, 2013). Placing the above-mentioned functions of PIs into perspective, it can be expected that regulation of angiogenesis and calcium mobilization will be more present at day 19 as compared to day 2.

Furthermore, days 2 and 9 could be distinguished from each other by the increased presence of CLs on day 2 as compared to a higher presence of PAs, PSs, and PEs on day 9 (Table 3). PA, a metabolite of phosphatidylcholine, plays a role in the mTOR signaling pathway, which acts as a pivotal nutrient sensing mechanism and partakes in regulating cellular differentiation, survival, growth, and division. Within this pathway, PA proposedly serves as an indicator for the presence of its lipid precursor molecules within the cell since these are important for the before-mentioned processes (Foster, 2013). Additionally, PA has shown to be an important component in the induction of angiogenesis through activating the HIF-1α-VEGF pathway (Han et al., 2014). Lastly, PA enhances inflammation through mTOR signaling, both *in vitro* as well as *in vivo* (Lim et al., 2003; Lee et al., 2007). The higher presence of PA on day 9 as compared to day 2 is in line with physiological fracture healing due to its roles in the regulation of cell fate and angiogenesis, as well as a more widespread, regulated inflammatory response that would more likely take place around day 9 after fracture as compared to day 2.

PEs, like PCs, are important precursor molecules of phosphoethanolamine, which on its turn is catalyzed by phosphoethanolamine/phosphocholine phosphatase (PHOSPHO1) to serve as a donor of inorganic phosphate in the production of hydroxyapatite. Furthermore, they are involved in the glycine and serine metabolism, two key amino acids in the production of collagen (Roberts et al., 2004). Increased presence of PEs on day 9, combined with relatively large scaled loadings, matches its role in both collagen metabolism, as well as a phosphate donor for hydroxyapatite production.

#### Lipid Patterns in Positive Ion Mode

In positive ion mode, day 2 and day 19 were mostly distinguishable by the presence of specific PCs on days 2 or 19. Furthermore, a higher presence of LPCs was observed on day 2 as compared to day 19 (Table 4).

PCs are amongst the most abundant phospholipids in mammalian cell membranes and are known to play important roles in cellular metabolism and energetic state (van der Veen et al., 2017). They also regulate endochondral bone formation by promoting chondrocyte differentiation and cartilage matrix degradation in growth plates (Li et al., 2014). Furthermore, PCs play a role in the glycine and serine metabolism, which are key amino acids in collagen synthesis, and act as precursors of phosphocholines, important inorganic phosphate donors in the production of hydroxyapatite and thus influence bone mineralization (Roberts et al., 2004; Albaugh et al., 2017; de Paz-Lugo et al., 2018). A study by Øyen *et al.* (2017) showed that dietary supplementation of choline, an essential nutrient that is predominantly supplied as PC, was directly associated with increased bone mineral density (Øyen et al., 2017). Since PCs are the most abundant phospholipids within mammalian cell membranes, it was to be expected that their presence in fxh would be validated on both days 2 and 19. However, it has yet to be revealed what causes the shift in specific lipid isoforms amongst the different time points. Unfortunately, information about the presence of specific lipids throughout fracture healing is lacking. The shift in specific PCs might be due to certain osteogenic processes that can be expected to be more present during day 19 as compared to day 2.

LPCs are known to elicit a seemingly contradictory effect on osteoclasts by inhibiting their formation whilst enhancing their function. Besides, although predominantly investigated in valvular calcification, LPCs enhance cellular mineralization (Mebarek et al., 2013; Wiltz et al., 2014; Bouchareb et al., 2015). The dual (opposite) effect of LPCs on both osteoclast formation and function could contribute to adequate osteoclast turnover and functionality in the early fracture environment.

Furthermore, day 9 could be separated from day 2 + 19 due to the higher presence of certain long chain CARs on day 2 + 19, whilst SM and LPC were more characteristic of day 9 (Table 4).

SMs are important cellular signaling molecules that play a role in bone formation and apoptosis. A deficiency in the enzyme that converts SM to PC has been shown to impair bone and cartilage mineralization (Khavandgar and Murshed, 2015). Additionally, a lack of SM due to the knockdown of sphingomyelin synthase 1 (SMS1) has been shown to impair bone development and case growth retardation due to the regulatory role of SMS1 in osteoblast development (Matsumoto et al., 2019). An increased presence of SM is, therefore, more likely to occur during day 9 after fracture since osteoblast development is initiated around that time (Bahney et al., 2019).

### Limitations and future Research

This study showed that MALDI-MSI allows for the identification of a fxh age-dependent lipid signature. Still, a larger sample cohort is needed to validate these data. Especially the lipid signature in fxh of fractures that develop impaired healing would be of interest. Due to the limited sample size and the fact that all fractures in our cohort healed without adverse events, no conclusions in relation to clinical outcome can be drawn at this stage. Theoretically, the fracture location within a bone could influence the presence of lipids in the fracture hematoma. However, since all included fractures were located in the metaphyseal bone, we expect this influence to be minimal. Moreover, analysis of metabolites or proteins, being key players in bone healing- and formation, could further provide insights into the molecular mechanisms in fracture healing. Future research should therefore focus on validating the results of the present study in a larger cohort and developing a method for analyzing the protein signature of the fxh.

## Conclusion

For the first time, our approach revealed the potential of MALDI-MSI as an analytical tool for the assessment of lipid patterns in fxh samples over time. An ammonium formate wash for 15 s twice was shown to adequately remove the heme from the porcine and human fxh and increase the overall molecular signal for negative and positive ion mode with minimum delocalization. The sample location within fxh is of less importance, as it was shown that the intra-variability within fxh was smaller than the inter-variability between fxh. Lastly, fxh age-dependent lipid patterns were identified, which showed to be involved in various processes that are important during fracture healing, such as angiogenesis, inflammation, cellular differentiation, mineralization, hydroxyapatite production, and collagen synthesis. Proper bone regeneration is in part dependent on the successful integration of the above-mentioned processes, and our results emphasize the important contributing role that lipids play. Assessment of specific molecular patterns within the fxh could provide insights into fracture healing processes and could potentially serve as a future source of theragnostic information on fracture healing.

## Data Availability

The raw data supporting the conclusion of this article will be made available by the authors, without undue reservation.
