# Supplementary material for: Lipid Analysis of Fracture Hematoma With MALDI-MSI: Specific Lipids are Associated to Bone Fracture Healing Over Time
Source: Front Chem. 2022 Mar 3;9:780626. doi: 10.3389/fchem.2021.780626 (PMC8927282; doi:10.3389/fchem.2021.780626)
Supplement: Supplementary file 1 [file DataSheet1.DOCX]

Supplementary Material


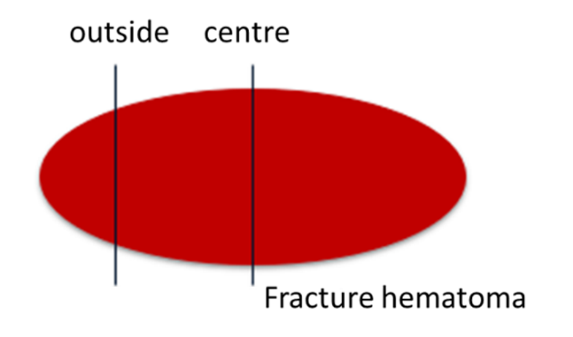


**Supplementary Figure S1: Schematic view of fxh and the location of sectioning for the outside and center sections.**


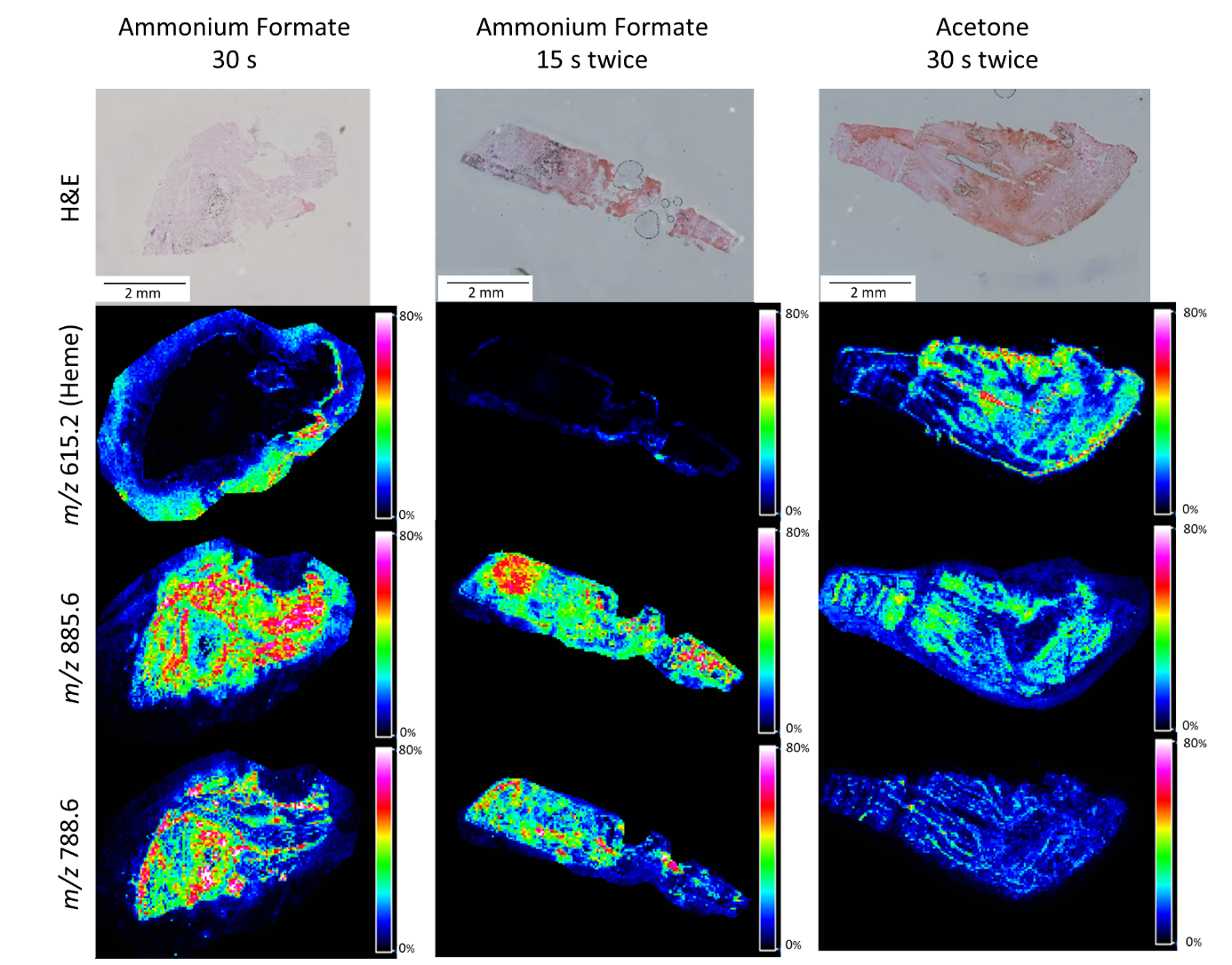


**Supplementary Figure S2: H&E stained and MALDI-MSI distribution images for three different washing methods for porcine fxh.** MALDI-MSI distribution images are shown for heme (*m/z* 615.2) and *m/z* values 885.6 and 788.6 in negative ion mode for the washing methods: ammonium formate for 30 seconds, ammonium formate for 15 seconds twice, and acetone for 30 seconds twice. All shown intensities are total ion current (TIC) normalized.


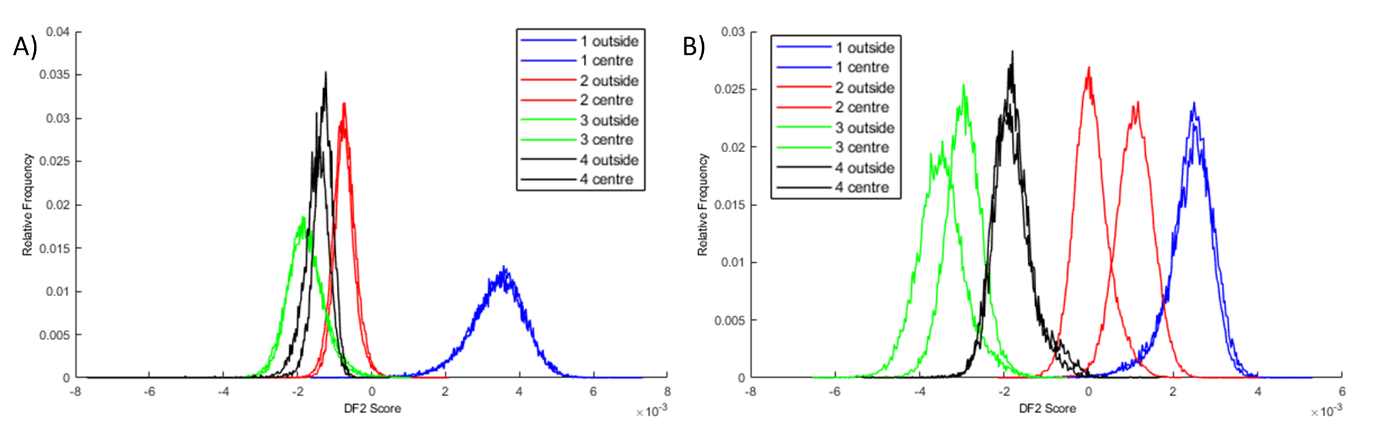


**Supplementary Figure S3: DF-2 scores for comparison of the intra-variability of fracture hematoma for negative and positive ion mode.** The DF-2 score explains the second biggest variance in the data set as determined by a PCA-LDA of the mass spectra of the outside and center sections of different porcine fxh. **A**) DF-2 score for the outside and center sections of four porcine fxh in negative ion mode. **B**) DF-2 score for the outside and center sections of four porcine fxh in positive ion mode.

**Supplementary Table S1**: **Comparison of the signal-to-noise (S/N) values for the different washing methods for negative and positive ion mode for porcine and human fracture hematoma (fxh)**.

**A**) S/N values for heme (*m/z* 615.2 = [Heme – H]^-^) and ten *m/z* values for the six washing methods and the control (no washing) for porcine fxh in negative ion mode. Wash 1 = Ammonium formate 15 sec twice; Wash 2 = Ammonium formate 15 seconds four times; Wash 3 = Ammonium formate 30 seconds; Wash 4 = Ammonium formate 30 seconds twice; Wash 5 = Acetone 30 seconds twice; Wash 6 = Acetone 30 seconds four times.

| *m/z* | 615.2 | 716.6 | 742.6 | 766.6 | 788.6 | 835.6 | 864.6 | 885.6 | 913.6 | 951.7 | 977.7 |
| --- | --- | --- | --- | --- | --- | --- | --- | --- | --- | --- | --- |
| No Wash | 7.1 | 15.0 | 7.5 | 11.0 | 300.8 | 188.8 | 20.4 | 67.6 | 21.5 | 69.3 | 21.4 |
| Wash 1 | 31.8 | 124.8 | 206.9 | 148.7 | 383.9 | 109.3 | 1051.8 | 1832.7 | 57.7 | 99.2 | 72.1 |
| Wash 2 | 42.0 | 41.8 | 70.5 | 31.2 | 142.5 | 140.9 | 1002.4 | 1581.6 | 66.9 | 87.5 | 74.8 |
| Wash 3 | 51.4 | 106.9 | 288.2 | 141.0 | 327.5 | 99.8 | 972.6 | 1839.4 | 73.5 | 42.8 | 28.7 |
| Wash 4 | 37.0 | 43.6 | 101.7 | 38.9 | 1177.6 | 117.6 | 895.3 | 1563.8 | 67.9 | 84.5 | 61.6 |
| Wash 5 | 717.0 | 61.1 | 196.0 | 79.1 | 168.6 | 45.2 | 385.0 | 920.6 | 37.5 | 2.0 | 0.0 |
| Wash 6 | 16.5 | 20.1 | 42.2 | 13.3 | 81.2 | 33.3 | 312.1 | 570.1 | 19.8 | 0.0 | 0.0 |

**B**) S/N values for heme (*m/z* 616.2 = [Heme]^+^) and ten *m/z* values for the three washing methods and the control (no washing) for porcine fxh in positive ion mode. Wash 1 = Ammonium formate 15 sec twice; Wash 2 = Ammonium formate 15 seconds four times; Wash 3 = Ammonium formate 30 seconds; Wash 4 = Ammonium formate 30 seconds twice; Wash 5 = Acetone 30 seconds twice; Wash 6 = Acetone 30 seconds four times.

| *m/z* | 616.2 | 650.5 | 734.6 | 760.6 | 786.6 | 808.6 | 810.6 | 824.6 | 887.7 | 901.7 | 927.7 |
| --- | --- | --- | --- | --- | --- | --- | --- | --- | --- | --- | --- |
| No Wash | 26.8 | 5.1 | 21.1 | 95.7 | 83.2 | 31.9 | 30.6 | 41.7 | 10.3 | 15.9 | 16.4 |
| Wash 1 | 11.7 | 152.6 | 270.1 | 1059.3 | 931.2 | 54.2 | 173.7 | 8.7 | 16.1 | 43.2 | 37.4 |
| Wash 3 | 600.8 | 125.0 | 195.4 | 1125.2 | 971.5 | 72.0 | 168.9 | 22.4 | 17.6 | 46.8 | 49.1 |
| Wash 5 | 381.6 | 56.8 | 174.3 | 358.3 | 320.8 | 86.0 | 85.2 | 117.1 | 0.0 | 0.0 | 0.0 |

**C**) S/N values for heme (*m/z* 615.2 = [Heme – H]^-^) and ten *m/z* values for the six washing methods and the control (no washing) for human fxh in negative ion mode. Wash 1 = Ammonium formate 15 sec twice; Wash 2 = Ammonium formate 15 seconds four times; Wash 3 = Ammonium formate 30 seconds; Wash 4 = Ammonium formate 30 seconds twice; Wash 5 = Acetone 30 seconds twice; Wash 6 = Acetone 30 seconds four times.

| *m/z* | 615.2 | 701.5 | 716.5 | 750.5 | 766.6 | 788.6 | 810.5 | 834.5 | 861.6 | 885.6 | 911.6 |
| --- | --- | --- | --- | --- | --- | --- | --- | --- | --- | --- | --- |
| No Wash | 254.6 | 0.0 | 13.4 | 4.2 | 5.8 | 26.0 | 27.1 | 10.4 | 13.0 | 46.5 | 0.0 |
| Wash 1 | 634.1 | 107.2 | 176.2 | 351.9 | 200.1 | 369.6 | 335.4 | 131.0 | 129.7 | 459.5 | 27.1 |
| Wash 2 | 60.6 | 48.3 | 46.8 | 36.8 | 34.2 | 107.6 | 115.9 | 51.2 | 75.6 | 305.6 | 14.4 |
| Wash 3 | 1008.0 | 108.9 | 149.8 | 260.3 | 149.6 | 259.9 | 437.0 | 176.0 | 128.3 | 527.6 | 23.1 |
| Wash 4 | 72.8 | 42.6 | 43.4 | 32.3 | 31.6 | 89.6 | 96.4 | 42.8 | 64.4 | 273.8 | 13.2 |
| Wash 5 | 1878.2 | 30.4 | 42.9 | 83.3 | 50.1 | 92.7 | 95.8 | 56.3 | 47.0 | 219.7 | 8.6 |
| Wash 6 | 846.7 | 9.1 | 11.9 | 7.4 | 8.6 | 22.7 | 25.4 | 8.9 | 11.7 | 40.2 | 0.0 |

**D**) S/N values for heme (*m/z* 616.2 = [Heme]^+^) and ten *m/z* values for the three washing methods and the control (no washing) for human fxh in positive ion mode. Wash 1 = Ammonium formate 15 sec twice; Wash 2 = Ammonium formate 15 seconds four times; Wash 3 = Ammonium formate 30 seconds; Wash 4 = Ammonium formate 30 seconds twice; Wash 5 = Acetone 30 seconds twice; Wash 6 = Acetone 30 seconds four times.

| *m/z* | 616.2 | 703.6 | 735.2 | 760.6 | 786.6 | 813.6 | 873.7 | 887.7 | 901.7 | 927.7 | 1025.8 |
| --- | --- | --- | --- | --- | --- | --- | --- | --- | --- | --- | --- |
| No Wash | 331.1 | 6.2 | 0.0 | 38.0 | 15.0 | 0.0 | 0.0 | 6.7 | 14.8 | 15.3 | 18.5 |
| Wash 1 | 436.1 | 460.6 | 55.7 | 1535.5 | 498.6 | 198.6 | 28.5 | 46.3 | 173.2 | 146.6 | 116.5 |
| Wash 3 | 1476.9 | 384.0 | 145.8 | 1349.9 | 492.3 | 173.9 | 26.1 | 47.8 | 168.6 | 146.8 | 101.5 |
| Wash 5 | 1707.2 | 53.3 | 67.5 | 236.0 | 93.4 | 29.1 | 0.0 | 0.0 | 0.0 | 0.0 | 0.0 |
